# Supplementary material for: Liver biopsy quality criteria to exclude cirrhosis in case of suspicion of porto-sinusoidal vascular disorder
Source: JHEP Rep. 2025 Nov 10;8(1):101670. doi: 10.1016/j.jhepr.2025.101670 (PMC12765425; doi:10.1016/j.jhepr.2025.101670)
Supplement: Multimedia component 1 [file mmc1.pdf]

Liver biopsy quality criteria to exclude cirrhosis in case of suspicion of porto-sinusoidal vascular disorder

Chloé de Broucker, Valérie Paradis, Maria Luisa Botero, Miguel Albuquerque, Audrey Payancé, Aurélie Plessier, Laure Elkrief, François Durand, Sophie Hillaire, Paul-Emile Zafar, Juan Carlos Garcia Pagan, Pierre-Emmanuel Rautou

Table of contents

Fig. S1..2

Fig. S2..3

Table S1..4

Fig. S1: Definition and examples of true positive, true negative, false positive, false negative and uninterpretable biopsy in this study.

	**Liver explant diagnosis**	
**Liver biopsy classification**	**Cirrhosis**	**PSVD**
**Cirrhosis**	True positive 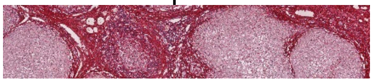	False positive 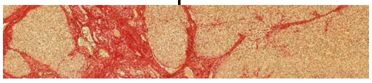
**No cirrhosis**	False negative 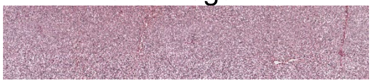	True negative 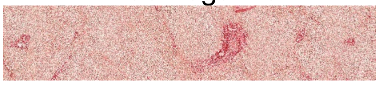
**Uninterpretable**	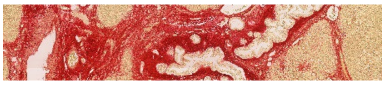	

PSVD, porto-sinusoidal vascular disorder

Fig. S2: Number of well-classified virtual liver biopsies with cirrhosis (*i.e.* sensitivity for the diagnosis of cirrhosis) according to the length and width of the virtual biopsy stained with picrosirius red.

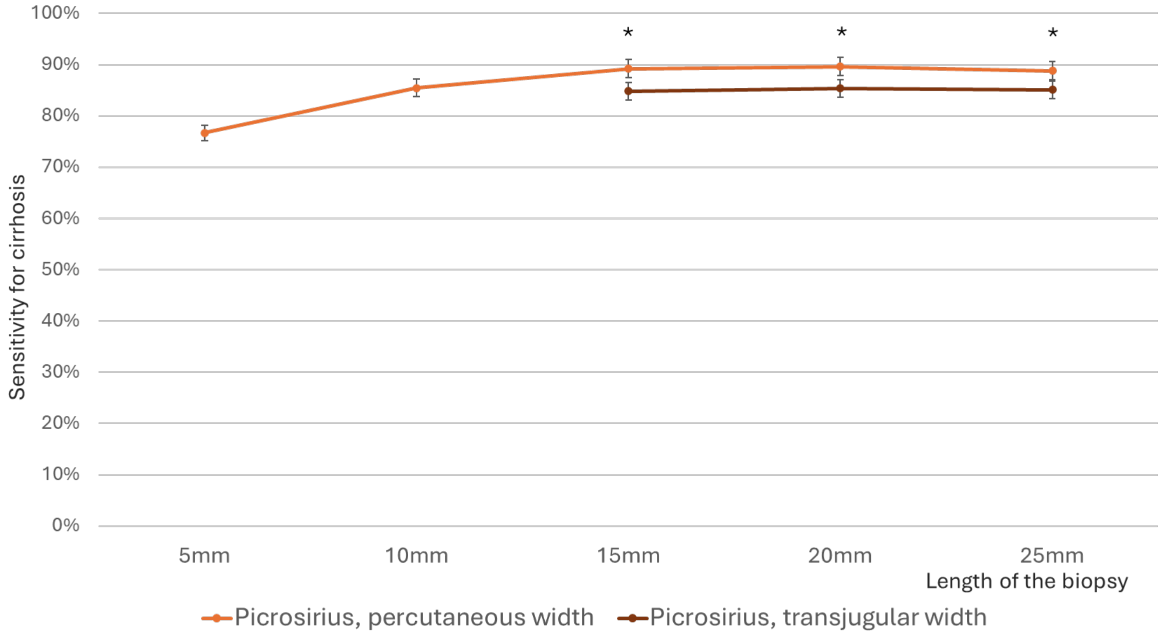

Sensitivity for cirrhosis is represented as a percentage and standard deviation. Groups were compared with a Chi2-test with a corrected risk α adapted with Bonferroni method: α'=α/4=0.0125. Sensitivity with transjugular route was significantly lower than percutaneous route in the length marked by a *.

Table S1: Sensitivity (%) for cirrhosis according to length, width and staining of the virtual liver biopsy

	**Staining**	Picrosirius red		Masson's trichrome
**Length**	**Width**<br>*Route mimicked*	1000 µm<br>*percutaneous*	572 µm<br>*transjugular*	1000µm<br>*percutaneous*
5 mm		77% (74-79%)		75% (72-77%)
10 mm		85% (84-87%)		82% (80-85%)
15 mm		89% (88-91%)<br>* 86% (84-88%)	85% (83-87%)	86% (84-88%)<br>* 86% (84-88%)
20 mm		90% (88-91%)	85% (83-87%)	88% (86-90%)
25 mm		89% (87-90%)	85% (83-87%)	87% (85-89%)
5+5+5 mm		85% (83-87%)		
5+10 mm		88% (86-90%)		

*, sensitivity with reader2.
