# Supplementary material for: Liver biopsy quality criteria to exclude cirrhosis in case of suspicion of porto-sinusoidal vascular disorder
Source: JHEP Rep. 2025 Nov 10;8(1):101670. doi: 10.1016/j.jhepr.2025.101670 (PMC12765425; doi:10.1016/j.jhepr.2025.101670)
Supplement: Multimedia component 2 [file mmc2.docx]

**Journal of Hepatology**

**CTAT methods**

Tables for a “Complete, Transparent, Accurate and Timely account” (CTAT) are now mandatory for all revised submissions. The aim is to enhance the reproducibility of methods.

- Only include the parts relevant to your study
- Refer to the CTAT in the main text as ‘Supplementary CTAT Table’
- Do not add subheadings
- Add as many rows as needed to include all information
- Only include one item per row

**If the CTAT form is not relevant to your study, please outline the reasons why:**

|  |
| --- |

- 1. **Antibodies**

**No antibodies were used in this study.**

- 1. **Cell lines**

**No cell lines were used in this study.**

- 1. **Organisms**

**No organisms were used in this study.**

- 1. **Sequence based reagents**

**No sequence based reagents were used in this study.**

- 1. **Biological samples**

| **Description** | **Source** | **Identifier** |
| --- | --- | --- |
| **Liver explants** | **Patients from Beaujon’s hospital** |  |

- 1. **Deposited data**

| **Name of repository** | **Identifier** | **Link** |
| --- | --- | --- |
|  |  |  |

- 1. **Software**

| **Software name** | **Manufacturer** | **Version** |
| --- | --- | --- |
| **SPSS 22.0** | **SPSS** | **22.0** |

- 1. **Other (e.g. drugs, proteins, vectors etc.)**

| **Picrosirius red staining** |  |  |
| --- | --- | --- |
| **Masson’s trichrome staining** |  |  |

- 1. **Please provide the details of the corresponding methods author for the manuscript:**

| Prof. Pierre-Emmanuel RAUTOU, MD, PhD  Service d’Hépatologie, Hôpital Beaujon, Assistance Publique des Hôpitaux de Paris,  100 Boulevard du Général Leclerc, 92110 Clichy, France  Telephone: +33 1 40 87 55 01  Fax: +33 1 40 87 55 30  E-mail: [pierre-emmanuel.rautou@inserm.fr](mailto:pierre-emmanuel.rautou@inserm.fr) |
| --- |

**2.0 Please confirm for randomised controlled trials all versions of the clinical protocol are included in the submission. These will be published online as supplementary information.**

| **This study is not a randomised controlled trial.** |
| --- |
