# Supplementary material for: Liver biopsy quality criteria to exclude cirrhosis in case of suspicion of porto-sinusoidal vascular disorder
Source: JHEP Rep. 2025 Nov 10;8(1):101670. doi: 10.1016/j.jhepr.2025.101670 (PMC12765425; doi:10.1016/j.jhepr.2025.101670)
Supplement: Multimedia component 4 [file mmc4.pdf]

Research article

JHEP Reports

Liver biopsy quality criteria to exclude cirrhosis in case of suspicion of porto-sinusoidal vascular disorder

Authors

Chloé de Broucker, Valérie Paradis, Maria Luisa Botero, …, Paul-Emile Zafar, Juan Carlos Garcia Pagan, Pierre-Emmanuel Rautou

Correspondence

pierre-emmanuel.rautou@inserm.fr (P.-E. Rautou), valerie.paradis@aphp.fr (V. Paradis).

Graphical abstract

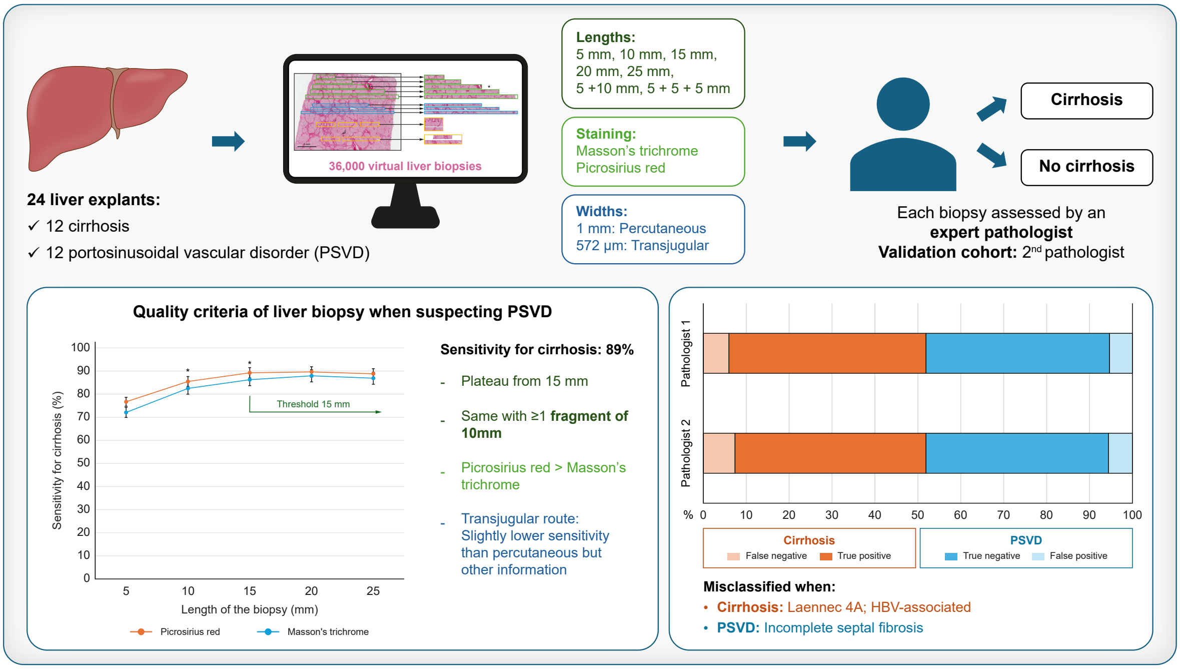

Highlights:

- Liver biopsies to rule out cirrhosis in PSVD should be ≥15 mm long with a ≥10 mm tissue fragment.
- Picrosirius red staining performs slightly better than Masson's trichrome to exclude cirrhosis.
- Percutaneous biopsies better exclude cirrhosis as they are wider than transjugular samples.
- Transjugular biopsy provides additional data on hepatic pressure gradient and vein-to-vein collaterals.
- Distinguishing incomplete septal cirrrhosis from LaennecA cirrhosis is difficult; repeat biopsy might be needed.

Impact and implications:

This study shows that, for the diagnosis of porto-sinusoidal vascular disorder, the minimum length of liver biopsy to exclude cirrhosis is 15 mm, with a minimum fragment of 10 mm. Picrosirius red had a slightly better performance than Masson's trichrome staining. Future guidelines might consider that a ≥15-mm-long biopsy, with a fragment ≥10 mm, is sufficient to rule out cirrhosis in case of suspicion of porto-sinusoidal vascular disorder with signs of portal hypertension.

https://doi.org/10.1016/j.jhepr.2025.101670
© 2025 The Author(s). Published by Elsevier B.V. on behalf of European Association for the Study of the Liver (EASL). This is an open access article under the CC BY license (http://creativecommons.org/licenses/by/4.0/). JHEP Reports, 2026, 8, 1–9

Liver biopsy quality criteria to exclude cirrhosis in case of suspicion of porto-sinusoidal vascular disorder

Chloé de Broucker[1,2], Valérie Paradis[1,3,*], Maria Luisa Botero[4,5,6,7], Miguel Albuquerque[1,3], Audrey Payancé[1,2], Aurélie Plessier[1,2], Laure Elkrief[8], François Durand[1,2], Sophie Hillaire[9], Paul-Emile Zafar[1,3], Juan Carlos Garcia Pagan[7], Pierre-Emmanuel Rautou[1,2,*]

JHEP Reports **2026**. vol. 8 | 1–9

Check for updates

Background & Aims: Baveno VII guidelines based porto-sinusoidal vascular disorder (PSVD) diagnosis on a liver biopsy excluding cirrhosis. However, evidence-based quality criteria for liver biopsy are lacking. This study aimed to determine biopsy length and staining appropriate to rule out cirrhosis.

Methods: Liver explants from 12 patients with cirrhosis and 12 with PSVD were selected. Slides were stained with Picrosirius red or Masson's trichrome. A total of 36,000 virtual liver biopsies were randomly generated, including different biopsy widths (572 and 1,000 μm corresponding to transjugular and percutaneous biopsies, respectively) and lengths (5 mm, 10 mm, 15 mm, 20 mm, 25 mm; fragmented 5 + 10 mm and 5 + 5 + 5 mm). Biopsies were assessed by an expert pathologist for the presence or absence of cirrhosis.

Results: Overall sensitivity of percutaneous biopsies for the diagnosis of cirrhosis was 85%, higher with Picrosirius red (86%) than with Masson's trichrome (83%) (p <0.001). Sensitivity increased with the length of percutaneous biopsies, reaching a plateau from 15 mm (88%). Sensitivity was significantly higher for percutaneous (89%) than for transjugular biopsies (84%) (p <0.001). A plateau was also observed from 15 mm for transjugular biopsies. Fragmented biopsies with at least one 10-mm-long fragment (5 + 10 mm) had similar sensitivity as 15-mm-long biopsies. Diagnostic accuracy was lower in case of Laennec A cirrhosis, HBV-associated disease, or incomplete septal fibrosis. Validation by a second pathologist gave similar results.

Conclusions: For the diagnosis of PSVD, the minimum length of liver biopsy to exclude cirrhosis was 15 mm with at least one fragment of 10 mm. Picrosirius red had a better performance than Masson's trichrome staining. The transjugular route showed lower sensitivity, but provides additional information.

© 2025 The Author(s). Published by Elsevier B.V. on behalf of European Association for the Study of the Liver (EASL). This is an open access article under the CC BY license (http://creativecommons.org/licenses/by/4.0/).

Introduction

Porto-sinusoidal vascular disorder (PSVD) is a broad clinico-pathological entity that includes several overlapping histological patterns (nodular regenerative hyperplasia, obliterative portal venopathy, hepatoportal sclerosis, and incomplete septal cirrhosis [ISF]) and clinical forms (non-cirrhotic portal fibrosis, idiopathic portal hypertension, and non-cirrhotic intrahepatic portal hypertension). PSVD is defined by the association of a liver biopsy excluding cirrhosis with clinical or radiological signs of portal hypertension, and/or histological lesions of PSVD.[1,2] Baveno VII guidelines recommend a minimum liver biopsy size of 20 mm with minimal fragmentation for the diagnosis of PSVD.[3] However, this recommendation is based on expert opinion only, as evidence on this topic is lacking.

The aim of this study was to define the quality criteria for liver biopsies to exclude cirrhosis in case of suspicion of PSVD.

Materials and methods

Endpoints

As the first aim of a liver biopsy in case of suspicion of PSVD is to rule out cirrhosis, the primary endpoint of this study was the sensitivity of the liver biopsy for the diagnosis of cirrhosis, that is, the proportion of biopsies from cirrhotic liver that were well classified as having cirrhosis that is, the true positives (Table S1). Tested criteria included length, fragmentation, staining for fibrosis, and width (to reflect the percutaneous *vs.* transjugular routes) of the biopsy.

* Corresponding authors. Addresses: Service d'Hépatologie, Hôpital Beaujon, Assistance Publique des Hôpitaux de Paris, 100 Boulevard du Général Leclerc, 92110 Clichy, France. Tel.: +33-1-40-87-55-01, Fax: +33-1-40-87-55-30 (P-E. Rautou); Service d'anatomo-pathologie, Hôpital Beaujon, Assistance Publique des Hôpitaux de Paris, 100 Boulevard du Général Leclerc, 92110 Clichy, France. Tel.: +33-1-40-87-54-59; Fax: +33-1-40-87-00-77 (V. Paradis).
E-mail addresses: pierre-emmanuel.rautou@inserm.fr (P.-E. Rautou), valerie.paradis@aphp.fr (V. Paradis).
https://doi.org/10.1016/j.jhepr.2025.101670

The secondary endpoint was the specificity of the liver biopsy for the diagnosis of cirrhosis, that is, the number of biopsies from PSVD liver well classified as having no cirrhosis, out of all biopsies with PSVD.

Patient selection

To obtain a large number of biopsies from patients with a definite diagnosis of PSVD or cirrhosis, we generated virtual biopsies from explants from patients with PSVD or cirrhosis, who underwent liver transplantation (LT). PSVD with signs of portal hypertension was defined according to Baveno VII as the association of a liver histology excluding cirrhosis with (a) one sign specific for portal hypertension (*i.e.* gastric, esophageal, or ectopic varices; portal hypertensive bleeding; portosystemic collaterals on imaging), or (b) one sign not specific for portal hypertension (ascites, platelet count <150 G/L or spleen size >13 cm) plus one histological lesion specific or non-specific for PSVD.[3] Twelve explants were retrospectively selected from a database of 36 patients with PSVD, who underwent LT between January 2008 and March 2022 at Beaujon Hospital, Clichy, France. Explants were selected using original histological reports. Diagnosis of PSVD was confirmed based on a second pathological examination of liver explants, combined with an analysis of clinical, laboratory, and imaging findings. To represent the main pathological lesions of PSVD, we selected four explants in which the main pathological lesion was nodular regenerative hyperplasia, four obliterative portal venopathy and four ISF. We also tried to balance the number of patients with each condition associated with PSVD.

We selected 12 explants out of the 123 explants from patients with cirrhosis attributable to metabolic dysfunction-associated steatohepatitis (MASH) and/or alcohol use disorder and/or HBV/HCV infection transplanted between 01 January 2020 and 01 April 2022 at Beaujon Hospital, Clichy, France. We balanced the number of patients with each cause of cirrhosis and also balanced the Laennec cirrhosis scores (4A, 4B and 4C).[4]

Generation of the virtual biopsies

Tissue samples measuring at least 2 × 2 cm from liver transplants were fixed in formalin and embedded in paraffin, and are hereafter referred to as ‘histological blocks’. A 3-μm-thick section was cut from one block per patient and stained with H&E, Picrosirius red, and Masson’s trichrome. Liver slides were digitized at 40 × (resolution: 1 pixel = 0.25 μm) using an APERIO GT450 DX scanner (LEICA©, Wetzlar, Germany) and the software provided by the manufacturer.

A script written in the laboratory was used to generate virtual biopsies of predefined widths and lengths from the digitized slides, as shown in Fig. 1. These biopsies were randomly distributed throughout the whole slide image and could be oriented horizontally or vertically. If necessary, two virtual biopsies could overlap. Virtual biopsies sometimes contained an area without tissue, typically when the virtual biopsy was taken from the sample periphery (see as an example the longest percutaneous biopsy in Fig. 1). None of the virtual liver biopsies had more than 20% of their surface area that contained no tissue. The virtual biopsies were then randomly assigned a 15-letter name to ensure pseudonymization. Image processing and computer operations were performed using Morphostar software (Imstar, Paris, France).

Variables

Table 1 summarizes all the variables compared in this study. A total of 100 biopsies were generated per patient, per length, per width, and per staining. H&E is the main stain for liver pathology, but it is not suitable for staging fibrosis.[5] Masson’s trichrome and Picrosirius red both stain extracellular matrix deposition, mainly collagens: blue with Masson’s trichrome and dark red with Picrosirius red. We compared these stains performed on the same histological block from consecutive sections. We compared five biopsy lengths: 5 mm, 10 mm, 15 mm, 20 mm, and 25 mm. To study the effect of a fragmentation on a liver biopsy, we compared 15-mm-long biopsies in a unique fragment to an association of (1) two biopsies of 10 and 5 mm, respectively, hereafter called ‘5 + 10 mm’, or (2) three 5-mm biopsies, hereafter called ‘5 + 5 + 5 mm’. We also compared biopsy width as the transjugular route requires thinner needles than percutaneous biopsies. To determine the width of the biopsies corresponding to each approach after fixation, we measured five widths at different sites of 20 percutaneous biopsies (performed using a 16-gauge needle) and of 20 transjugular biopsies (performed using a 19-gauge Tru-cut needle). The mean width of the biopsies was 936 μm (standard deviation: 161 μm) for the percutaneous approach and 535 μm (standard deviation: 165 μm) for the transjugular approach. For the sake of simplicity, we used 1 mm and 572 μm (proportionally calculated) width when creating the virtual biopsies, to represent percutaneous and transjugular approaches, respectively. Each individual biopsy stain (Picrosirius red and Masson’s trichrome) was examined as a separate batch. As Picrosirius red staining yielded better results than Masson’s trichrome (see Results), only the Picrosirius red was used for the analysis of transjugular biopsies.

Analysis of the biopsies

Pseudonymized liver biopsies were assessed for the presence or absence of cirrhosis by a single pathologist (VP). This pathologist routinely uses Picrosirius red staining to assess liver fibrosis. Cirrhosis was defined as the presence of two or more hepatocellular nodules surrounded by fibrous bands. Biopsies were classified as ‘uninterpretable’ when large portal tracts or areas of parenchymal extinction occupied a significant proportion of the biopsy surface. For statistical analyses, biopsies classified as ‘uninterpretable’ were counted as misdiagnosed.

Validation by an independent reader

A validation was performed by a second pathologist from another center (MLB) who examined some of the same biopsies (n = 4,800). This pathologist routinely uses Masson’s trichrome to assess liver fibrosis. Before validation began, both pathologists read together a random selection of 400 biopsies, independent from the validation cohort, including different biopsy lengths, widths, and stains. They agreed on the definition of cirrhosis and of uninterpretable biopsies, as described above.

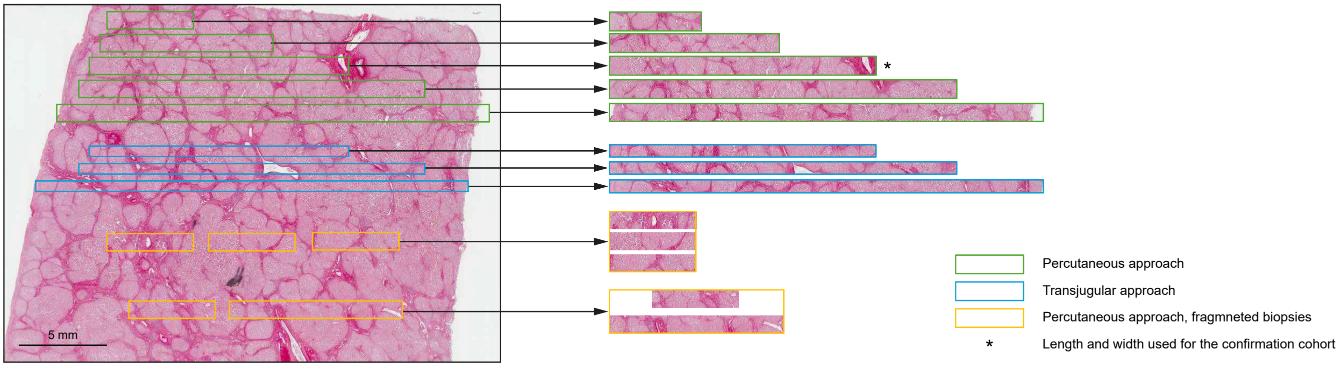

Fig. 1. Example of generated virtual liver biopsies from a digitized pathological slide derived from a liver explant with cirrhosis. The virtual biopsies circled in green represent percutaneous biopsies; those circled in blue represent transjugular biopsies and those circled in yellow represent fragmented percutaneous biopsies. From the top to the bottom, virtual liver biopsies are: 5-mm-long percutaneous biopsy, 10-mm-long percutaneous biopsy, 15-mm-long percutaneous biopsy, 20-mm-long percutaneous biopsy, 25-mm-long percutaneous biopsy, 15-mm-long transjugular biopsy, 20-mm-long transjugular biopsy, 25-mm-long transjugular biopsy, 5 + 5 + 5-mm-long fragmented percutaneous biopsy, 5 + 10-mm-long fragmented percutaneous biopsy.

IRB approval

The study was conducted in accordance with the ethical guidelines of the 1975 Declaration of Helsinki and was approved by the institutional review board (CPP Sud Méditerranée V, Nice, France – Ethics approval number 2021-A02148-33). Informed consent was obtained from all individuals.

Statistical analysis

Categorical variables were expressed as number of patients n (%, 95% CI) and compared using the X^2 test. Quantitative variables were expressed as the median and IQR and were compared using a Student's *t* test or a Mann-Whitney *U* test, as appropriate.

All tests were performed two-sided and with a first-order risk adjusted according to the Bonferroni correction, as the primary endpoint included four variables (biopsy length, width, staining, and fragmentation). All tests were done with a corrected alpha risk of: $\alpha' = \alpha/4 = 0.0125$. We used the Cochran–Armitage test for trend to compare sensitivity according to the biopsy length and the Friedman test to compare staining and width according to the biopsy length. Statistical analyses were performed using SPSS version 22.0 software (SPSS Inc., Chicago, IL, USA). All authors had access to the study data, and reviewed and approved the final manuscript.

Results

Liver explants selected

Characteristics of the 12 patients with cirrhosis and the 12 patients with PSVD selected are summarized in Table 2. In patients with cirrhosis, Laennec scores were evenly distributed among 4A, 4B and 4C. Causes of cirrhosis were MASH (n = 6), alcohol-related liver disease (n = 6), HBV infection (n = 2), and HCV infection (n = 2) (four patients had several causes of cirrhosis). Both patients with a history of HCV infection had achieved sustained virological response following treatment >5 years before LT; HCV RNA was undetectable for both of them at the time of LT. Four patients had hepatocellular carcinoma (HCC). Patients with PSVD had the following associated conditions: cystic fibrosis (n = 3), HIV infection (n = 2), *TERT* gene mutation (n = 1), heterozygous prothrombin gene mutation (n = 1), antiphospholipid syndrome associated with protein S deficiency (n = 1), Sjögren's syndrome and hypogammaglobulinemia (n = 1), systemic lupus erythematosus (n = 1), common variable immunodeficiency (CVID) (n = 1), and none (n = 1). All the patients had at least one histological lesion specific for PSVD and at least one clinical sign specific to PSVD. Histological analysis of the livers of patients with PSVD showed obliterative portal venopathy (n = 12), nodular regenerative hyperplasia (n = 5), and incomplete septal cirrhosis (n = 6) (all patients had several lesions of PSVD).

Sensitivity for cirrhosis

The 36,000 virtual biopsies generated (*i.e.* the 15 conditions depicted in Table 1, for 12 cirrhosis and 12 PSVD patients) were assessed by a unique pathologist for the presence or absence of cirrhosis.

Out of these 36,000 virtual biopsies generated, 484/36,000 virtual liver biopsies were classified as 'uninterpretable'. Biopsies were more often classified as 'uninterpretable' when they were 5 mm long, as compared with longer biopsies [2.6% (1.9–3.2%) *vs.* 0.4% (0.3–0.5%) respectively, p <0.001], and when biopsies were generated from patients with PSVD as compared to cirrhosis [2.1% (1.9–2.3%) and 0.6% (0.5–0.7%) respectively, p <0.001].

We first assessed sensitivity for cirrhosis, that is, number of true positive in patients with cirrhosis, according to biopsy staining and length, using 1-mm-wide biopsies, representing percutaneous liver biopsies (Fig. 2 and Table S1). Overall sensitivity for the diagnosis of cirrhosis was 85% (84–85%). This sensitivity was higher with Picrosirius red than Masson's trichrome staining [86% (95% CI 85–87%) *vs.* 83% (95% CI 82–84%), p <0.001]. Using Friedman test by length, Picrosirius red also had higher sensitivity than Masson's trichrome (p <0.001). Regarding length of biopsies, sensitivity for cirrhosis increased with biopsy length for both stains (p <0.001 using a Cochran–Armitage test for trend). Yet, sensitivity reached a plateau at 15 mm, at which point sensitivity was 89% (88–91%) for Picrosirius red and 86% (84–88%) for Masson's trichrome (Table S1).

Table 1. Number of generated virtual liver biopsies per liver explant, according to length, width and staining.

Staining	Picrosirius red		Masson's trichrome
Width, route mimicked	1,000 µm, percutaneous	572 µm, transjugular	1,000 µm, percutaneous
Length			
5 mm	100		100
10 mm	100		100
15 mm	100*	100*	100
20 mm	100	100	100
25 mm	100	100	100
5 + 5 + 5 mm	100		
5 + 10 mm	100		

'5 + 5 + 5 mm' represented fragmented liver biopsies with three fragments of 5 mm each. '5 + 10 mm' represented fragmented liver biopsies with two fragments of 5 mm and 10 mm *Biopsies used for the validation cohort.

We compared biopsy widths obtained using transjugular *vs.* percutaneous route. As we did not expect better sensitivity with thinner biopsies, we compared transjugular and percutaneous width only for ≥15-mm-long biopsies using Picrosirius red staining (Fig. S2). Overall, transjugular biopsies had a lower sensitivity for cirrhosis than percutaneous biopsies [85% (95% CI 84–86%) *vs.* 89% (95% CI 88–91%), respectively (p <0.001)]. This difference remained when analyzing each length separately. For transjugular biopsies, just like percutaneous biopsies, a plateau was observed from 15 mm.

Table 2. Clinical characteristics of the patients at the time of liver transplantation.

	Cirrhosis		Patients with PSVD	
Clinical features		n =		n =
Male sex (n)	8	12	6	12
Age at time of liver transplantation (years)	62 (56-64)	12	53 (43-59)	12
BMI (kg/m^2)	27 (24-32)	12	24 (22-27)	12
Complications of the liver disease				
Ascites (n)	7	12	8	12
Spontaneous ascites infection (n)	1	12	3	12
Pleural effusion (n)	0	12	2	12
Portal vein thrombosis (n)	5	12	7	12
History of portal hypertensive bleeding (n)	4	12	6	12
Hepatic encephalopathy (n)	4	12	5	12
Hepatorenal syndrome (n)	0	12	0	12
Porto-pulmonary hypertension (n)	1	12	0	12
Hepatopulmonary syndrome	0	12	2	12
Alcohol-related hepatitis (n)	2	12	0	12
Hepatocellular carcinoma (n)	4	12	0	12
Drugs				
Anticoagulation (n)	2	12	5	12
Diuretics (n)	7	12	8	12
Beta-blockers (n)	7	12	5	12
Endoscopic data				
Esophageal varices (n)	6	12	10	12
Hemodynamic data				
Hepatic pressure venous gradient (mmHg)	21 (16–23)	12	12 (12–16)	5
Imaging data				
Porto-systemic collaterals at imaging (n)	10	12	10	12
Splenomegaly (n)	6	12	11	12
Partial occlusion of the portal vein (n)	2	12	7	12
Laboratory data				
Hemoglobin (g/dl)	10 (8–11)	12	10.2 (8.5–12.1)	11
White blood cell count (g/L)	5 (3–7)	12	4.7 (3.3–7.8)	12
Platelet count (g/L)	91 (79–104)	12	88 (73–135)	12
Prothrombin index (%)	55 (30–68)	12	74 (64–93)	12
International normalized ratio	1.3 (1.3–2.3)	12	1.19 (1.08–1.36)	12
Factor V (%)	54 (27–71)	12	57 (53–77)	10
Serum alanine aminotransferase (IU/L)	53 (20–32)	12	35 (32–65)	12
Serum aspartate aminotransferase (IU/L)	24 (21–32)	12	36 (27–56)	12
Serum alkaline phosphatase (IU/L)	100 (76–164)	12	185 (127–218)	12
Serum gamma glutamyltransferase (IU/L)	51 (32–113)	12	56 (33–108)	12
Serum bilirubin (µmol/L)	41 (22–145)	12	21 (12–33)	12
Serum sodium (mmol/L)	135 (134–138)	12	138 (136–139)	12
Serum creatinine (µmol/L)	87 (69–105)	12	71 (57–76)	12
Serum albumin (g/L)	30 (26–35)	12	26 (21–31)	10
MELD score	**17 (11–23)**	12	**10 (6–12)**	12

Quantitative variables are expressed in median (IQR). Qualitative variables are expressed in number of patients (n). MELD, model for end-stage liver disease; PSVD, porto-sinusoidal vascular disorder.

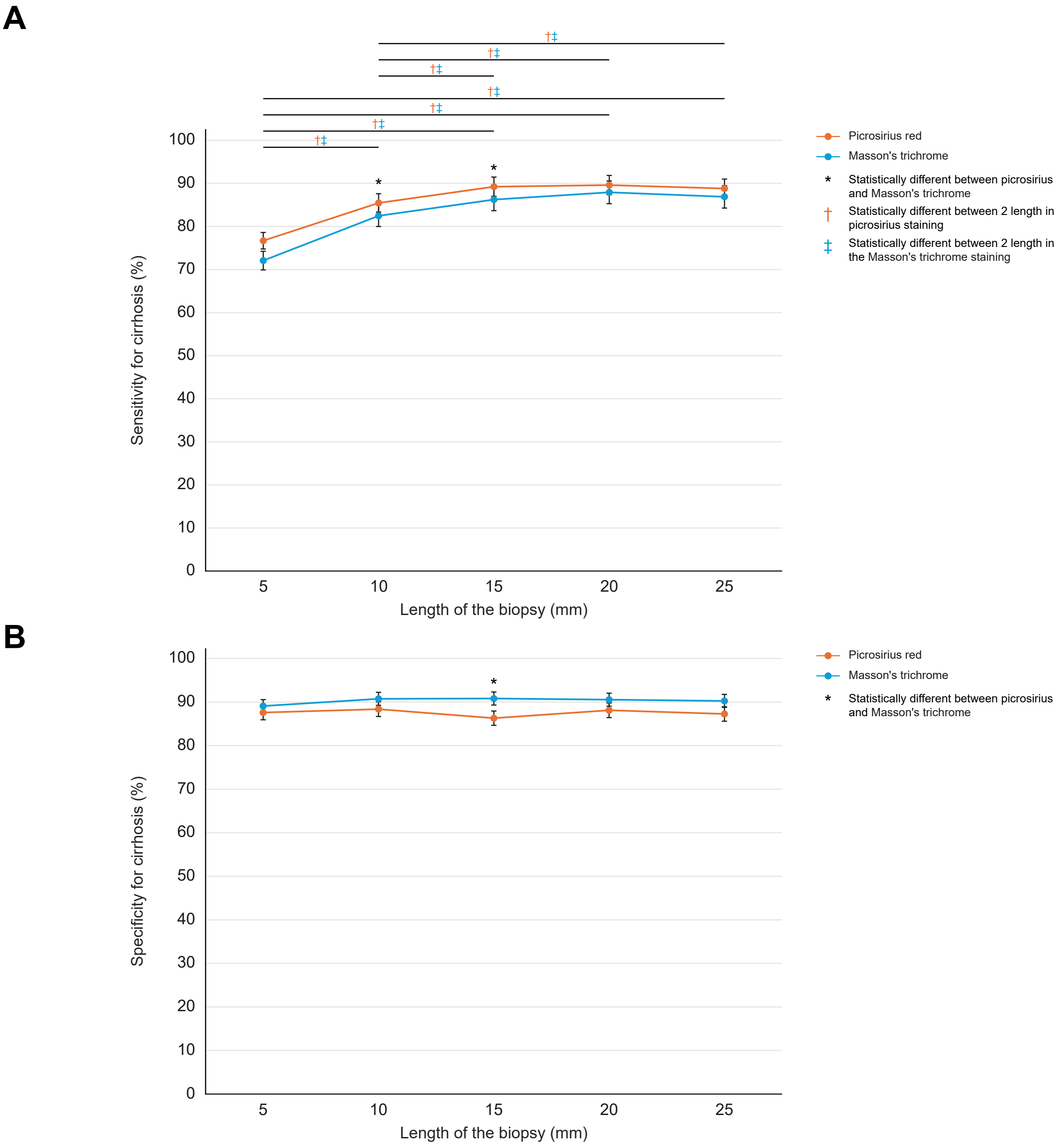

Fig. 2. Sensitivity and specificity for the diagnosis of cirrhosis according to length and staining of 1-mm-wide virtual biopsy. (A) Sensitivity and (B) specificity for cirrhosis are represented as a percentage and standard deviation. Groups were compared with a X^2 test with a corrected risk α adapted with the Bonferroni method: $\alpha' = \alpha/4 = 0.0125$. *Shows differences statistically significant between Picrosirius red and Masson's trichrome. †Shows differences statistically significant between two lengths in Picrosirius red staining. ‡Shows differences statistically significant between two lengths in Masson's trichrome staining.

We then assessed the impact of liver biopsy fragmentation. We observed that the sensitivity for cirrhosis of the '5 + 5 + 5 mm' biopsies (84% [95% CI 83–87%]) was significantly lower than that of the '5 + 10 mm' biopsies (88% [95% CI 86–90%], $p = 0.01$] and of the 15 mm biopsies (89% [95% CI 88–91%], $p < 0.001$]. However, sensitivity was not significantly different between '5 + 10 mm' and 15-mm-long biopsies.

We investigated in more details the impact of cirrhosis stage and cause on sensitivity for cirrhosis. As shown in Fig. 3A and B, sensitivity for cirrhosis was systematically above 90% in patients with Laennec score 4B or 4C cirrhosis, when the biopsy was 15 mm or more in length. This sensitivity was much lower for Laennec 4A cirrhosis (59% [95% CI 58–60%], $p < 0.001$). Besides, biopsies from liver explants with HBV-related cirrhosis had significantly lower sensitivity for cirrhosis (73% [95% CI 72–75%]) than biopsies from liver explants with other causes of cirrhosis (87% [95% CI 87–88%], $p < 0.001$) (Fig. 3A and B). However, in HBV-related cirrhosis,

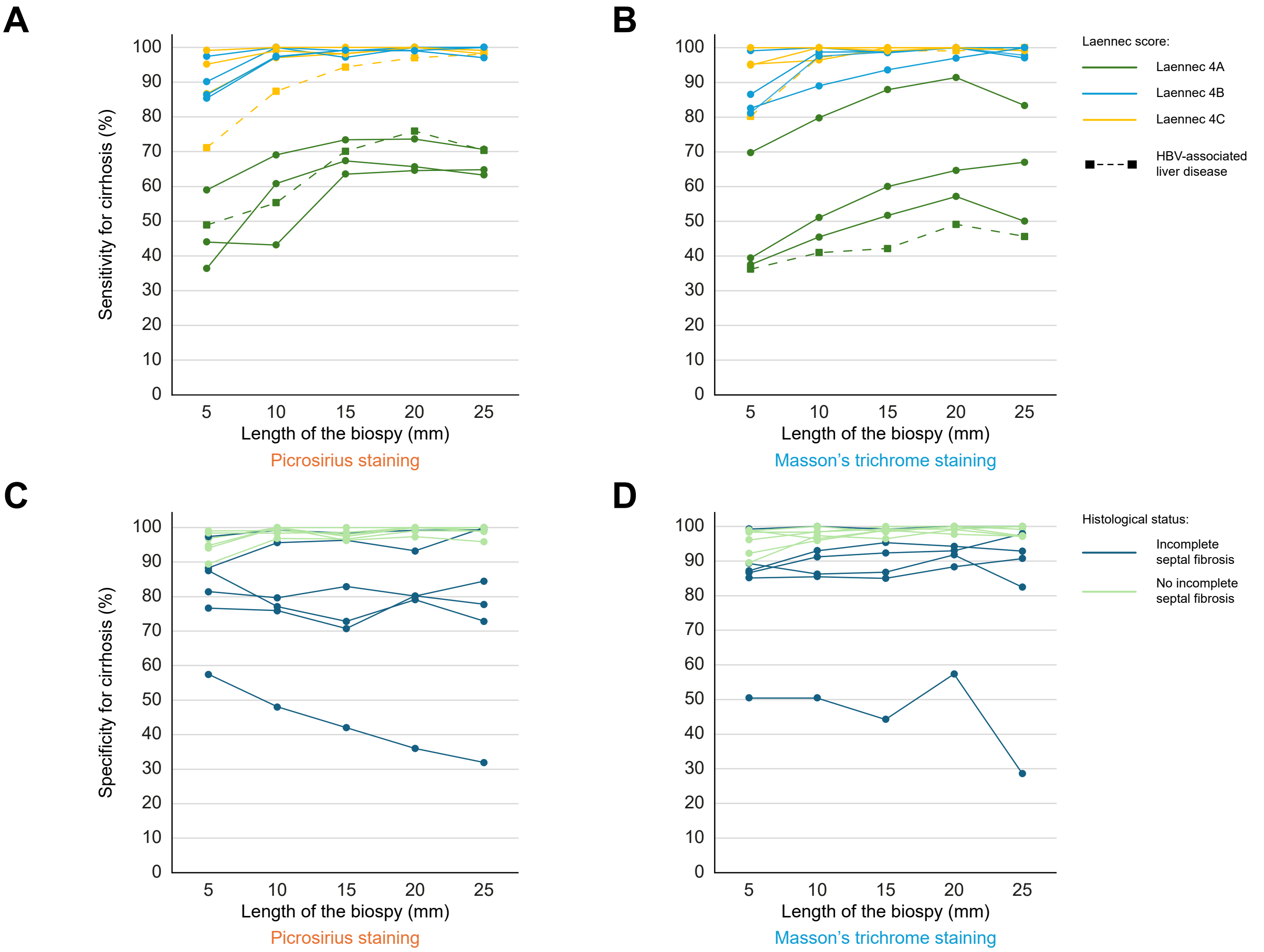

Fig. 3. Sensitivity and specificity for the diagnosis of cirrhosis according to the length, the staining of the virtual biopsy and the Laennec score. Sensitivity for the diagnosis of cirrhosis according to the length, the staining of the virtual biopsy and the Laennec score, using Picrosirius red staining (A) and Masson's trichrome staining (B). Each line represents the explant from a patient with cirrhosis (n = 12). Specificity for cirrhosis according to the length and the staining of the virtual biopsy and to the presence or absence of incomplete septal cirrhosis on the liver explant, using Picrosirius red staining (C) and Masson's trichrome staining (D). Each line represents the explant from a patient with PSVD (n = 12).

there was no significant difference in sensitivity for cirrhosis between biopsies of 15, 20 or 25 mm (data not shown). Out of the two liver explants from patients with HBV infection, one was Laennec 4A and one was Laennec 4C.

Accuracy of the liver biopsy and specificity for cirrhosis

Accuracy of the virtual liver biopsies, that is the proportion of biopsies that were correctly classified was 87% (86–87%) with Picrosirius red and 87% (86–88%) with Masson's trichrome. It also reached a plateau from 15 mm length, and was slightly lower with transjugular biopsies (86% [85–87%], p <0.001].

Specificity for cirrhosis is the proportion of virtual biopsies from PSVD liver explants that were classified as 'non-cirrhotic' out of all PSVD biopsies. Overall, specificity was slightly lower for Picrosirius red than Masson's trichrome staining (88% [86–91%] *vs.* 90% [86–92%], respectively; p <0.001). These proportions did not improve with biopsy length (Fig. 2B). We then investigated in more detail the impact of PSVD histological lesions and observed that specificity was lower in cases of PSVD with ISF *vs.* PSVD without ISF (80% [79–81%] *vs.* 98% [98–98%], respectively, p <0.001). Similar results were obtained using Masson's trichrome (Fig. 3C and D).

Validation by an independent reader

For the independent validation, a second pathologist read 15-mm percutaneous biopsies only, as these were associated with the best sensitivity for cirrhosis according to the first pathologist. Biopsies with both stains were examined, leading to a total of 4,800 virtual biopsies. Overall sensitivity for cirrhosis in the independent validation was 86% (84–87%) (Fig. 4). While Reader one routinely uses Picrosirius red staining to stage fibrosis, Reader two uses Masson's trichrome. According to Reader two's assessment, sensitivity for cirrhosis did not differ between Picrosirius red and Masson's trichrome staining (86% [84–88%] and 86 [84–89%], respectively; p = 0.907); specificity neither differed between Picrosirius red and Masson's trichrome (88% [86–90%] and 90% [87-92%], respectively, p = 0.163)]. Fig. 4 illustrates the true and false positive and negative rates for each pathologist.

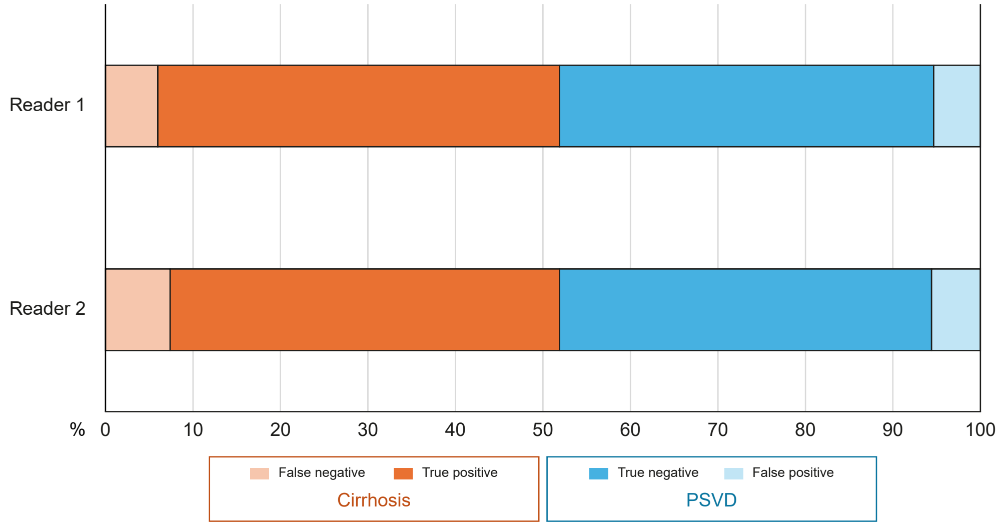

Fig. 4. Proportion of well-classified virtual percutaneous 15-mm-long liver biopsies, using Picrosirius red staining, according to the pathologist. PSVD, porto-sinusoidal vascular disorder.

Discussion

PSVD is a rare condition, the definition of which includes a good quality liver biopsy to exclude cirrhosis. By analyzing 36,000 virtual liver biopsies, this study provides evidence-based information on liver biopsy quality criteria in case of suspicion of PSVD.

The first major finding of this study is that a 15 mm biopsy can rule out cirrhosis in case of suspicion of PSVD, with a sensitivity of 89%. Beyond this threshold of 15 mm, the diagnostic performance of the liver biopsy to exclude cirrhosis reaches a plateau. This plateau was observed for both Picrosirius red and Masson's trichrome staining, and for width representing both percutaneous and transjugular biopsies, making the data consistent. Similar diagnostic performance was observed when fragmented biopsies had at least one fragment measuring 10 mm. A minimum biopsy length of 20 mm was recommended by Baveno VII guidelines,[3,6,7] based on studies evaluating fibrosis scoring in chronic viral hepatitis.[5,8] Future guidelines might change that statement and consider that a biopsy of at least 15 mm in length with a fragment of at least a 10 mm in length is sufficient to rule out cirrhosis in case of suspicion of PSVD with signs of portal hypertension. A longer biopsy might still be needed to identify lesions of PSVD (*e.g.* portal vein stenosis, nodular regenerative hyperplasia), in particular in patients without signs of portal hypertension in whom PSVD diagnosis relies on histology only.[9,10] It should be noted that the minimum biopsy length of 15 mm mentioned here is after fixation. As the size of the biopsy sample reduces slightly after fixation and processing, physicians performing biopsies should aim for a slightly larger sample size.[11]

The second finding is that Picrosirius red performed slightly better than Masson's trichrome. While these two stains highlight extracellular matrix, mostly collagens, some pathologists are more accustomed to one stain than the other. We therefore deliberately chose one pathologist routinely using Picrosirius red and the other Masson's trichrome, to take experience into account. In the derivation cohort, Picrosirius red had a higher sensitivity for cirrhosis than Masson's trichrome. In the validation study, while the pathologist routinely uses Masson's trichrome, the sensitivity for cirrhosis was similar with Masson's trichrome than with Picrosirius red. Therefore, although differences are mild, Picrosirius red might be preferred to exclude cirrhosis while suspecting PSVD.

We also investigated route of the liver biopsy. We observed that biopsies performed with 16-gauge needles (typical for percutaneous liver biopsies) had a better sensitivity for cirrhosis than 19-gauge needles (typical for Tru-Cut transjugular biopsies). However, in practice, transjugular biopsy is recommended in patients with low platelets count (<50 G/L) or anticoagulant therapy, for example for portal vein thrombosis, which is common in PSVD.[12–14] Moreover, the transjugular route provides additional information useful for PSVD diagnosis, including hepatic venous pressure gradient and the presence of hepatic vein-to-vein collaterals, which might compensate for the slightly lower specificity for cirrhosis (85% *vs.* 89%).[12,15]

We identified situations in which sensitivity for cirrhosis is lower. These include patients with Laennec A cirrhosis or HBV-related cirrhosis, consistent with previous data from the literature.[16] Indeed, it is known that different causes of cirrhosis lead to different degrees and patterns of fibrosis: HBV-related cirrhosis is more often macronodular and some cases of ISF have been described.[17] Practically speaking, in case of suspicion of PSVD with HBV infection, macronodular cirrhosis should be ruled out.[18] We also identified that diagnostic accuracy was lower in PSVD cases with ISF. Baveno VII guidelines stated: '*Incomplete septal fibrosis (also called incomplete septal cirrhosis) can only be assessed on liver explants and not on liver biopsies*'. Our findings support maintaining this statement in the Baveno VIII recommendations. The diagnosis of ISF must be made with caution and may be questioned until LT.

In the present study, all patients with PSVD required LT, mainly because of complications of portal hypertension, and thus had a severe liver disease. Even if robust data on the

evolution of histological lesions of PSVD over time are lacking, Hercun *et al.*[19] recently reported that liver fibrosis progresses with time in patients with PSVD and CVID. This progression of liver fibrosis may account for the higher proportion of ISF in the present study (50%) than reported in recent studies (8–17%).[12,20] If this increase in liver fibrosis over time is a general feature of PSVD (*i.e.* not restricted to PSVD associated with CVID), the sensitivity of 15 mm biopsies for diagnosing cirrhosis would be even higher than the 88% reported here. Indeed, with less fibrosis on liver biopsies from patients with PSVD, diagnostic accuracy would have been even higher than that reported here. Of note, features of cirrhosis are not entirely specific, as they were observed in a small proportion of well-characterized patients with PSVD.

This study has several limitations: (i) 12 cases of cirrhosis and 12 of PSVD might not be sufficient to sample the variety of histological changes that may be seen[21]; (ii) multiple samples taken from a single liver are not truly independent; (iii) 25-mm-long virtual biopsies sometimes required to associate two fragments, as the liver samples were not large enough; (iv) only the most common causes of cirrhosis were represented in this study; (v) this study focused on excluding cirrhosis, leaving other questions around the diagnosis of PSVD for further studies. The choice of having as many cirrhosis and PSVD samples was arbitrary, but might not be far from a typical clinical scenario. Indeed, while the prevalence of cirrhosis is much higher than that of PSVD, in practice, biopsy is performed in a small minority of the patients, typically in case of low liver stiffness measurement values and/or absence of cause of cirrhosis. In the near future, artificial intelligence may be able to rule out cirrhosis based on liver biopsies. Further research is needed to explore this approach.

In conclusion, this study shows that for the diagnosis of PSVD, the minimum length of liver biopsy to exclude cirrhosis is 15 mm, with a minimum fragment of 10 mm. Picrosirius red had a slightly better performance than Masson's trichrome staining. Sensitivity of transjugular biopsy is slightly lower than percutaneous, but the transjugular route provides other useful information, including the hepatic venous pressure gradient and the presence or absence of hepatic vein-to-vein collaterals. However, if there is a high suspicion of PSVD or cirrhosis and the biopsy result does not match the clinical suspicion, a second biopsy should be considered, especially if HBV infection is present or if histology suggests Laennec A cirrhosis or ISF.

These data may be considered in the forthcoming Baveno VIII recommendations.

Affiliations

[1]Université Paris-Cité, Inserm, Centre de recherche sur l'inflammation, UMR 1149, Paris, France; [2]AP-HP, Hôpital Beaujon, Service d'Hépatologie, DMU DIGEST, Centre de Référence des Maladies Vasculaires du Foie, FILFOIE, ERN RARE-LIVER, Clichy, France; [3]Département de pathologie, hôpital Beaujon, AP-HP Nord, Clichy, France; [4]Barcelona Pathology Consulting Services, Barcelona, Spain; [5]Hepatic Hemodynamic Laboratory, Liver Unit, Hospital Clínic, Clínic Barcelona, Health Care Provider of the European Reference Network on Rare Liver Disorders (ERN-RareLiver), CSUR Centro de referencia del Sistema Nacional de Salud en Enfermedad Hepática Vascular Compleja en adultos. Fundació de Recerca Clínic Barcelona-Institut d'Investigacions Biomèdiques August Pi i Sunyer (FRCB-IDIBAPS), Barcelona, Spain; [6]Centro de Investigación Biomédica en red Enfermedades Hepáticas y Digestivas (CIBEREHD), Madrid, Spain; [7]AGAUR SGR2021 01115. Medicine Department, Faculty of Medicine, and Health Science, Universitat de Barcelona, Barcelona, Catalonia, Spain; [8]CHRU de Tours-Hôpital Trousseau, Service d'Hépato-Gastroentérologie, Centre de référence Constitutif Maladies vasculaires du foie, FILFOIE, ERN RARE-LIVER Faculté de Médecine de Tours, Tours, France; [9]Hôpital Foch, Médecine Interne, Suresnes, France

Abbreviations

CVID, common variable immunodeficiency; HCC, hepatocellular carcinoma; ISF, incomplete septal fibrosis; LT, liver transplantation; MASH, metabolic dysfunction-associated steatotic hepatitis; MELD, model for end-stage liver disease; PSVD, porto-sinusoidal vascular disorder.

Financial support

This article is based upon work from COST Action EURO-VALDI-NET, CA23146, supported by COST (European Cooperation in Science and Technology). This work has been funded by the "bourse FARE (Fonds d'Aide à la Recherche et à l'Evaluation)" provided by the SNFGE. P-E.R.'s research laboratory is supported by the Fondation pour la Recherche Médicale (FRM EQU202303016287), "Institut National de la Santé et de la Recherche Médicale" (ATIP AVENIR), the "Agence Nationale pour la Recherche" (ANR-18-CE14-0006-01, RHU QUID-NASH, ANR-18-IDEX-0001, ANR-22-CE14-0002) by «Émergence, Ville de Paris», by Fondation ARC, by the European Union's Horizon 2020 research and innovation programme under grant agreement No 847949 (DECISION) and No. 825575 (RiTa) and by France 2030 RHU LIVER-TRACK (ANR-23-RHUS-0014).

Conflicts of interest

P-ER has received research funding from Terrafirma and acted as consultant for Mursla, Genfit, Boehringer Ingelheim, Cook, AstraZeneca, Jazz and Abbelight, and received speaker fees from AbbVie.

Please refer to the accompanying ICMJE disclosure forms for further details.

Authors' contributions

Study concept and design: CDB, JCG-P, VP, P-ER. Funding acquisition: LE, P-ER. Acquisition of data: FD, CDB, SH, AP, AP. Followed-up and recorded patient information: P-ER. Produced the virtual biopsies: MA, P-EZ. Read the biopsies: MLB, VP. Data analysis: CDB, P-ER. Supervision: CDB, JCGP, VP, P-ER. Drafting the manuscript: CDB, P-ER. Critical review of the manuscript: all authors.

Data availability

The authors are prepared to provide the data from this study upon request to the corresponding author.

Acknowledgements

We thank Marie Santin, Kamal Zekrini and Djalila Seghier for their help in collecting the data and Nathalie Colnot for her help with liver biopsy samples.

Supplementary data

Supplementary data to this article can be found online at https://doi.org/10.1016/j.jhepr.2025.101670.

References

Author names in bold designate shared co-first authorship

[1] De Gottardi A, Sempoux C, Berzigotti A. Porto-sinusoidal vascular disorder. J Hepatol 2022;77:1124–1135.

[2] De Gottardi A, Rautou PE, Schouten J, et al. Porto-sinusoidal vascular disease: proposal and description of a novel entity. Lancet Gastroenterol Hepatol 2019;4:399–411.

[3] De Franchis R, Bosch J, Garcia-Tsao G, et al. Baveno VII – renewing consensus in portal hypertension. J Hepatol 2022;76:959–974.

[4] Kutami R, Girgrah N, Wanless IR, et al. The Laennec grading system for assessment of hepatic fibrosis: validation by correlation with wedged hepatic vein pressure and clinical features. Hepatology 2000;57:556–563.

[5] Cholongitas E, Senzolo M, Standish R, et al. A systematic review of the quality of liver biopsy specimens. Am J Clin Pathol 2006;125:710–721.
[6] Neuberger J, Patel J, Caldwell H, et al. Guidelines on the use of liver biopsy in clinical practice from the British society of gastroenterology, the royal college of radiologists and the royal college of pathology. Gut 2020;69:1382–1403.
[7] Rockey DC, Caldwell SH, Goodman ZD, et al. Liver biopsy. Hepatology 2009;49:1017–1044.
[8] Bedossa P, Dargère D, Paradis V. Sampling variability of liver fibrosis in chronic hepatitis C. Hepatology 2003;38:1449–1457.
[9] Guido M, Alves VAF, Balabaud C, et al. Histology of portal vascular changes associated with idiopathic non-cirrhotic portal hypertension: nomenclature and definition. Histopathology 2019;74:219–226.
[10] Cazals-Hatem D, Hillaire S, Rudler M, et al. Obliterative portal venopathy: portal hypertension is not always present at diagnosis. J Hepatol 2011;54: 455–461.
[11] Riley TR, Ruggiero FM. The effect of processing on liver biopsy core size. Dig Dis Sci 2008;53:2775–2777.
[12] **Magaz M**, **Giudicelli-Lett H**, Abraldes JG, et al. Porto-sinusoidal vascular liver disorder with portal hypertension: natural history and long-term outcome. J Hepatol 2025;82:72–83.
[13] Riescher-Tuczkiewicz A, Caldwell SH, Kamath PS, et al. Bleeding in Liver Diseases Investigators. Expert opinion on bleeding risk from invasive procedures in cirrhosis. JHEP Rep 2024;6:100986.
[14] Riescher-Tuczkiewicz A, Rautou PE. Prediction and prevention of post-procedural bleedings in patients with cirrhosis. Clin Mol Hepatol 2025;31 (Suppl):S205–S227.
[15] Kalambokis G, Manousou P, Vibhakorn S, et al. Transjugular liver biopsy – indications, adequacy, quality of specimens, and complications – a systematic review. J Hepatol 2007;47:284–294.
[16] Helmreich-Becker I, Schirmacher P, Denzer U, et al. Minilaparoscopy in the diagnosis of cirrhosis: superiority in patients with Child-Pugh A and macronodular disease. Endoscopy 2003;35:55–60.
[17] Wanless IR, Nakashima E, Sherman M. Regression of human cirrhosis. Arch Pathol Lab Med 2000;124:1599–1607.
[18] Olivas P, Perez-Campuzano V, Orts L, et al. Porto-sinusoidal vascular disorder in chronic HBV: a significant coexistence not to be overlooked. JHEP Rep 2024;6:100996.
[19] Hercun J, Asif B, Vittal A, et al. Development of hepatic fibrosis in common variable immunodeficiency-related porto-sinusoidal vascular disorder. Aliment Pharmacol Ther 2024;60:888–896.
[20] Wöran K, Semmler G, Jachs M, et al. Clinical course of porto-sinusoidal vascular disease is distinct from idiopathic noncirrhotic portal hypertension. Clin Gastroenterol Hepatol 2022;20:e251–e266.
[21] Ciriaci N, Bertin L, Rautou PE. Genetic predisposition to porto-sinusoidal vascular disorder. Hepatology 2024. https://doi.org/10.1097/HEP.0000000000000973.

Keywords: Histology; Pathology; Nodular regenerative hyperplasia; Obliterative portal venopathy.
Received 24 July 2025; received in revised form 29 October 2025; accepted 30 October 2025; Available online 10 November 2025

Journal of Hepatology, Volume *8*

Supplemental information

Liver biopsy quality criteria to exclude cirrhosis in case of suspicion of porto-sinusoidal vascular disorder

Chloé de Broucker, Valérie Paradis, Maria Luisa Botero, Miguel Albuquerque, Audrey Payancé, Aurélie Plessier, Laure Elkrief, François Durand, Sophie Hillaire, Paul-Emile Zafar, Juan Carlos Garcia Pagan, and Pierre-Emmanuel Rautou

Liver biopsy quality criteria to exclude cirrhosis in case of suspicion of porto-sinusoidal vascular disorder

Chloé de Broucker, Valérie Paradis, Maria Luisa Botero, Miguel Albuquerque, Audrey Payancé, Aurélie Plessier, Laure Elkrief, François Durand, Sophie Hillaire, Paul-Emile Zafar, Juan Carlos Garcia Pagan, Pierre-Emmanuel Rautou

Table of contents

Fig. S1 2

Fig. S2 3

Table S1 4

Fig. S1: Definition and examples of true positive, true negative, false positive, false negative and uninterpretable biopsy in this study.

	**Liver explant diagnosis**	
**Liver biopsy classification**	**Cirrhosis**	**PSVD**
**Cirrhosis**	True positive 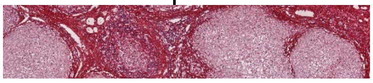	False positive 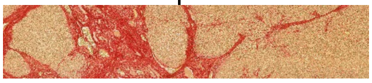
**No cirrhosis**	False negative 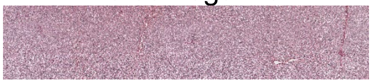	True negative 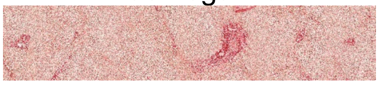
**Uninterpretable**	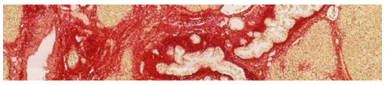	

PSVD, porto-sinusoidal vascular disorder

Fig. S2: Number of well-classified virtual liver biopsies with cirrhosis (*i.e.* sensitivity for the diagnosis of cirrhosis) according to the length and width of the virtual biopsy stained with picrosirius red.

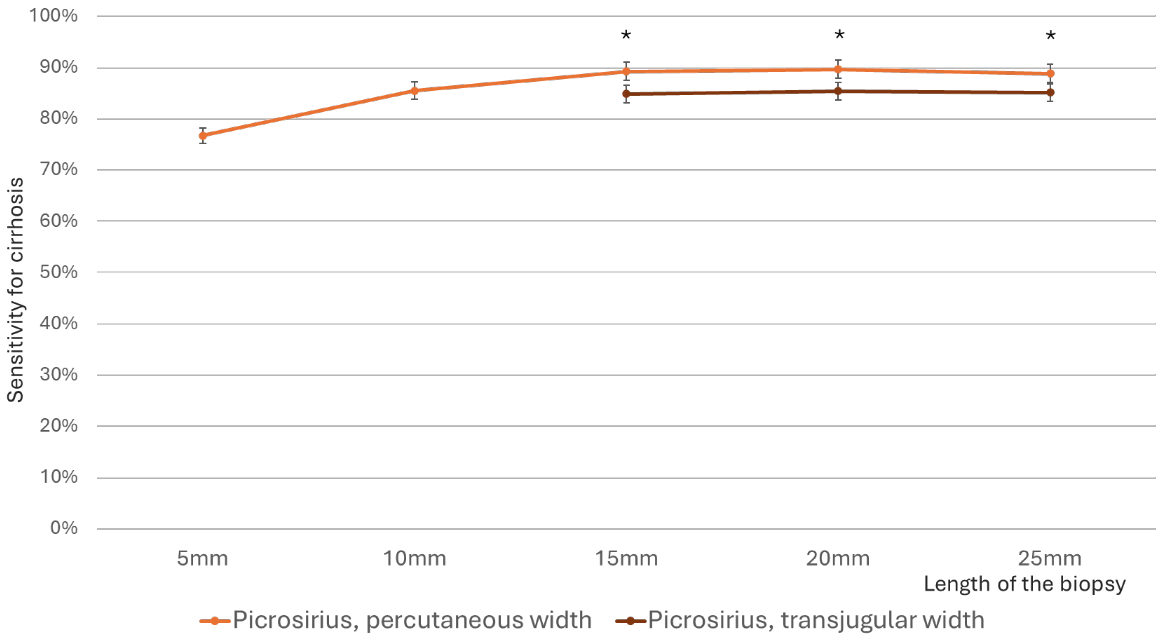

Sensitivity for cirrhosis is represented as a percentage and standard deviation. Groups were compared with a Chi2-test with a corrected risk α adapted with Bonferroni method: α'=α/4=0.0125. Sensitivity with transjugular route was significantly lower than percutaneous route in the length marked by a *.

Table S1: Sensitivity (%) for cirrhosis according to length, width and staining of the virtual liver biopsy

	**Staining**	Picrosirius red		Masson's trichrome
**Length**	**Width**<br>*Route mimicked*	1000 µm<br>*percutaneous*	572 µm<br>*transjugular*	1000µm<br>*percutaneous*
5 mm		77% (74-79%)		75% (72-77%)
10 mm		85% (84-87%)		82% (80-85%)
15 mm		89% (88-91%)<br>* 86% (84-88%)	85% (83-87%)	86% (84-88%)<br>* 86% (84-88%)
20 mm		90% (88-91%)	85% (83-87%)	88% (86-90%)
25 mm		89% (87-90%)	85% (83-87%)	87% (85-89%)
5+5+5 mm		85% (83-87%)		
5+10 mm		88% (86-90%)		

*, sensitivity with reader2.
